# Photothermal Sensitive 3D Printed Biodegradable Polyester Scaffolds with Polydopamine Coating for Bone Tissue Engineering

**DOI:** 10.3390/polym15020381

**Published:** 2023-01-11

**Authors:** Zuoxun Huang, Junfeng Li, Xiaohu Chen, Qing Yang, Xiyang Zeng, Ruqing Bai, Li Wang

**Affiliations:** 1 College of Materials, Chemistry & Chemical Engineering, Chengdu University of Technology, Chengdu 610059, China; 2 State Key Laboratory of Mechanical Transmission, Chongqing University, Chongqing 400044, China; 3 Department of Biomedical Engineering, School of Big Health and Intelligent Engineering, Chengdu Medical College, Chengdu 610500, China

**Keywords:** 3D printing, polylactic acid, tissue engineering, photothermal, polydopamine coating

## Abstract

Biodegradable scaffolds with photothermal effects and customizable pore structures are a hot topic of research in the field of bone repair. In this study, we prepared porous scaffolds using poly(lactic acid) (PLA) as the raw material and customized the pore structure with 3D printing technology. First, we investigated the effect of pore structure on the mechanical properties of this 3D PLA scaffold. Subsequently, the optimally designed PLA scaffolds were coated with PDA to enhance their hydrophilicity and bioactivity. XRD (X-ray diffraction), FTIR (Fourier transform infrared spectroscopy) and EDS (Energy dispersive spectroscopy) results indicated that PDA was successfully coated on the surface of PLA scaffolds. SEM (Scanning electron microscopy) micrographs showed that the surface of the PDA/PLA scaffolds became rough. WCA (water contact angle) confirmed that the material has enhanced hydrophilic properties. PDA/PLA scaffolds exhibit a tunable photothermal effect under NIR (near infrared) irradiation. The 3D-printed PLA/PDA scaffolds have remarkable potential as an alternative material for repairing bone defects.

## 1. Introduction

Bone disorders are of significant concern due to an increase in the median age of the population [1]. In daily life, severe trauma and accidents can lead to severe bone defects, resulting in severe disability that greatly interferes with the patient’s life quality [2]. To solve this problem, bone tissue engineering scaffolds have attracted considerable attention because they can provide extracellular matrix and structural support for bone reconstruction and regeneration [3]. Developing a type of bone scaffold with excellent osteoinductivity and programmable pore structure is the current research focus [4]. Several advanced and efficient manufacturing methods are used to manufacture porous biomaterials, such as self-assembly [5,6] and electrospinning [7]. However, most methods for obtaining scaffolds suffer from disadvantages such as poor pore structure and interconnectivity, long subsequent processing steps, and difficulty in rapidly preparing patient-specific scaffolds [8,9,10]. Compared with other methods, the most significant advantage of 3D printing technology based on fused deposition modeling (FDM) is the ability to fabricate custom shapes with an interior lattice network connecting them. They could provide a personalized porous structure that traditional manufacturing techniques cannot adequately attribute, which allows it can programmatically edit and design bone scaffolds with porous structures that can be precisely shaped. These structures are being used for direct implantation into the human body in the biomedical field in areas such as bio-printing, where this potential is being heavily utilized [11]. FDM works by the extrusion of small beads or polymer filament in the form of a molten thermoplastic from a small nozzle, which then hardens post-printing to form a solid construct [12]. Controllable variables include raster thickness, raster gap width, and raster angle, which can be adjusted to fabricate scaffolds with controlled pore size, morphology, and interconnectivity [13]. Therefore, the FDM process can produce complex 3D structures that are difficult to create by other traditional manufacturing methods such as lithography and micromachining, which also means that the use of 3D printing technology can target different bone defects in patients with bone diseases [14]. Customized manufacturing ensures that the implanted bone scaffold fits the patient’s bone defect area to the greatest extent possible [15]. Many studies have demonstrated that these FDM-based scaffolds have favorable mechanical and biochemical properties for bone regeneration [16,17,18]. Meanwhile, the literature reveals that properties of FDM parts are functions of various process-related parameters and can be significantly improved with proper adjustment [19]. Therefore, the printing precision and mechanical properties of scaffolds by adjusting the parameters of the layer thickness, filling rate, printing speed, and nozzle temperature were critical in past bone regeneration studies [20,21].

Appropriate biodegradability is also an essential characteristic for a scaffold to provide sufficient mechanical support while the new bone is forming and to prevent triggering an inflammatory response toward the foreign material of the scaffold [15]. Poly(lactic acid) (PLA) is a biodegradable polymer that has been approved by the Food and Drug Administration (FDA) and is used for various biomedical applications [22], which is one of the most commonly used polymers for FDM due to advantages such as biocompatibility, biodegradability, and low cost. The melting point temperature of this material makes it suitable for forming filaments and it can be extruded at a temperature of 180 –250℃ [23]. Gandolfi et al. prepared poly(lactic acid)-based porous scaffolds with no toxic effect, which showed excellent biodegradability [24]. Meanwhile, PLA can mimic well the mechanical properties of natural bone. Gremare et al. produced poly(lactic acid) (PLA) scaffolds of different pore sizes using a fused deposition modeling (FDM) technique and evaluated their physicochemical and biological properties, which did not exhibit any cytotoxicity toward human bone marrow stromal cells (HBMSCs) and showed excellent biocompatibility [25]. However, pure PLA is a typical hydrophobic polymer material that lacks cell recognition signals and has limited applications in biomaterials [26]. To overcome these limitations, it is suggested to enhance its hydrophilicity and bioactivity by means of surface modification [27]. Hydrophilic PDA with a large amount of catechol structure gives it superb adhesion and can easily coat the PLA surface [28]. The PDA coating is generated by the oxidative self-polymerization of PLA by soaking it in dopamine (DA) under specific conditions, without the need for complex processes.

After bone scaffold implantation, bacterial infection, the postoperative inflammatory response and potentially causing tumor-producing lesions to affect the health of patients. Photothermal therapy (PTT) is a typical photon-triggered therapy that kills bacteria or tumor cells by local heating generated by a photothermal agent (PTA) under visible or near-infrared (NIR) light [29]. Currently available PTAs mainly focus on Au-, Ag-, and Pd-based novel metal nanoparticles [30,31], Cu-based semiconductor nanoparticles [32], carbon-based nanomaterials [33], and organic polymers [34]. Despite efficient cancer therapy, these agents have not yet achieved clinical implementation, stemming from great concerns regarding their long-term safety. While, PDA has excellent biocompatibility and photothermal effect which allow it can produce heat under irradiation with near-infrared light. Compared with other PTAs, PDA can degrade in the human body and therefore has better biosafety [35]. It has been demonstrated that the cancer cells can be killed after maintenance at 42 °C for 15–60 min [36], this duration can be shortened within 2 min for temperatures over 50 °C. Hence, through the photothermal effect of PDA, it can effectively avoid bacterial infection during surgery, and the inflammatory reaction after surgery and can be used in cancer photothermal therapy [37].

**Figure 1:**
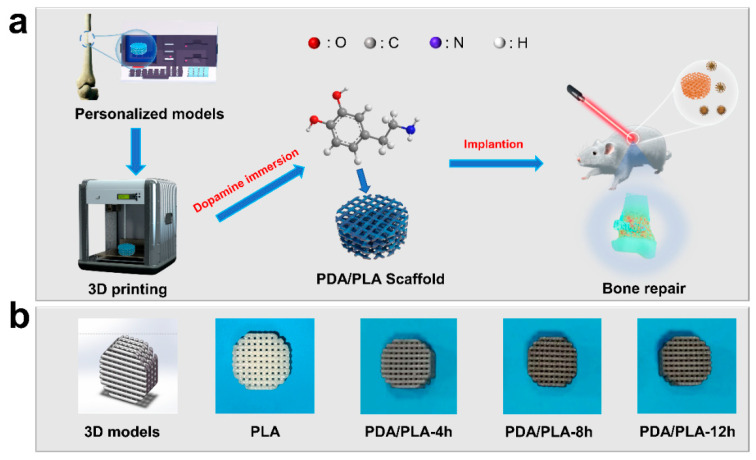
(**a**) Preparation process of PDA/PLA scaffolds, (**b**) 3D models of scaffolds and photographs of scaffolds prepared by immersion in PDA for different times.

In this work, we aimed to develop a customized biomimetic design for the internal structure of the patient’s bone defect area, therefore, a 3D porous bone scaffold model that is suitable for cell adhesion to the transmission was designed and fabricated using PLA as polymer matrix by 3D printing. To enhance the bioactivity of bone scaffolds and further explore more surface modifications, we adopted the abovementioned approach to coat poly(dopamine) on PLA scaffolds (Figure 1a). The chemical composition, morphology, microstructure, and wettability of PDA/PLA porous scaffolds were characterized and analyzed. The biocompatibility of the materials was evaluated by in vitro degradation and in vitro mineralization. Near-infrared irradiation was used to evaluate the photothermal effect of the scaffolds. This material is expected to be an alternative material for bone tissue engineering scaffolds.

## 2. Experimental

### 2.1. Materials

Dopamine hydrochloride (98%) was purchased from Aladdin (Shanghai, China). Tris(hydroxymethyl) aminomethane (Tris base) (CAS NO. 77-86-1) was purchased from Scientific Phygene (Fuzhou, China). Printable PLA wire materials for 3D printing were obtained from Hori (Beijing, China), and hydrochloric acid was acquired from Chron Chemicals (Chengdu, China).

### 2.2. Preparation of PLA/PDA Scaffolds

Three-dimensional models were first designed using Solidworks 2012, and then the Hori Z300 plus 3D printer (Hori, China) was used to fabricate 3D scaffolds. In the 3D printing machine, a cartridge was used to supply the feedstock filament of PLA (Hori, China) into the fused deposition modeling (FDM) 3D printer.

3-Hydroxytyramine hydrochloride (dopamine hydrochloride) was dissolved in 10 mM Tris buffer (pH 8.5) to 1 mg mL^−1^. All scaffolds were rinsed with deionized water before poly(dopamine) deposition. Polymer scaffolds were soaked in 100 μL of dopamine solution at room temperature for various periods. Meanwhile, the scaffolds were stirred under light-proof and airtight conditions. The prepared scaffolds were named PDA/PLA-xh, where x stands for the duration of immersion.

### 2.3. Physio-Chemical Characterization

Fourier transform infrared (FTIR) analysis was performed in the wavenumber range of 400–4000 cm^−1^ with 64 scans per spectrum at 4 cm^−1^ resolution (FTIR, TENSOR-27, Bruker, Germany). Wide angle X-ray scattering (WAXS) was carried out with a step size of 0.07° at a scanning speed of 0.3° s^−1^ with Cu-Kα radiation in the 2θ range of 5–70° using an X-ray diffractometer (XRD) (DX-2700, China Fangyuan Instrument Co., Ltd., Hongkong, China) operating at 40 kV and 25 mA. The microstructure morphology of the composite scaffolds was analyzed by field emission scanning electron microscopy (FE-SEM, INSPECTF50, FEI, Holland) at an accelerating voltage of 10 kV. Mechanical testing: The mechanical properties of porous composite scaffolds were determined using an electronic universal testing machine (MIT-30KN, Changzhou SFMIT apparatus Co., Ltd., Changzhou, China), and scaffold samples (10 mm × 10 mm × 5 mm) with the same composition were subjected to each test at room temperature, 65% RH and a crosshead speed of 1.0 mm min^−1^ to obtain an average value. The contact angle can be used to measure the hydrophilicity of the material. The contact angle of the sample was measured by an SDC-200 contact angle tester (Dongguan Shengding Precision Instrument Co., Ltd., Ruian, China). The material was made into a 40 mm × 40 mm × 5 mm sample, 2 μL water droplets were deposited on the surface of the material using deionized water as the medium, and images were captured by the tester. Three parallel samples were set for each sample, and their average value was taken.

### 2.4. Degradation In Vitro

Degradation test composites were determined by quantification of the mass loss of samples (three different pore size scaffolds) after incubation in PBS at body temperature (37 °C). The medium was exchanged for PBS solution every 6 days to prevent saturation. At the same time, the samples were removed, rinsed with distilled water, weighed after drying at 60 °C for 1 day, and returned to the medium after weighing. The liquor change, drying, and weighing processes were repeated three days at a time. Each group of samples was independently analyzed in three experiments, and the test results were averaged. The degradation rate of the scaffold was calculated with the following formula: $W_{L}=\frac{W_{0-}W_{1}}{W_{0}}\times 100\%$, where *W*_0_ represents the initial weight of the material and *W*_1_ represents the remaining weight of the sample at different times.

### 2.5. Mineralization In Vitro

Simulated body fluid (SBF, pH = 7.4) was used to evaluate the mineralization behavior of porous composite scaffolds in vitro. Each porous PLA/PDA scaffold with different soaking times was soaked in 20 mL of SBF and then placed in a 37 °C water bath for 7 days. After the immersion ended, the porous composite scaffolds were removed from the SBF and dried under a vacuum for SEM observation. 

### 2.6. Near Infrared Test

A near-infrared laser transmitter was used to examine the photothermal conversion effect of PLA/PDA scaffolds with different immersion times. These NIR experiments were performed using a laser transmitter with a wavelength of 808 nm (HW808AD2000-34F, Shenzhen Infrared Laser Technology Co., Ltd., Shenzhen, China). The irradiation distance was 0.3 m, and the spot size was 2 cm.

### 2.7. Statistical Analysis

All data are expressed as the means ± standard deviations (SD) with Origin 2021 (OriginLab, Northampton, MA, USA). A Student’s *t*-test was used to assess the statistical significance of the data between groups, and a value of *p* < 0.05 was considered to be statistically significant.

## 3. Results and Discussion

### 3.1. Analysis of the Optimal Pore Structure

**Figure 2:**
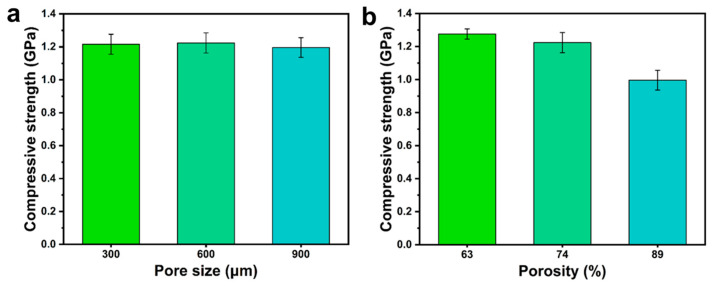
(**a**) Compressive strength of PLA scaffolds (74% porosity) with different pore sizes, (**b**) Compressive strength of PLA scaffolds (600 μm) with different porosity.

Bone tissue engineering scaffolds require appropriate mechanical properties to provide mechanical support [38]. However, the microstructure (porosity and pore size) of scaffolds has a nonnegligible effect on their mechanical properties [39]. Therefore, the effect of the pore structure of the scaffold on the performance was studied before the PDA-modified PLA scaffold. Figure 2 shows that the different pore sizes all presented high mechanical properties, and the pore size has little effect on the compressive strength of the scaffolds. But it has been reported that the pore size of scaffolds has a large effect on the proliferation and adhesion of cells. For instance, Cove et al. designed PLA porous scaffolds with pore sizes of 300, 600, and 900 µm and functionalized the scaffolds with a collagen coating. After seven days, the scaffolds with pore sizes of 600 µm exhibited relatively higher cell proliferation and adhesion than those with pore sizes of 300 and 900 μm [40]. Inspired by this work, the pore size of the porous scaffolds was determined to be 600 µm. High porosity scaffolds can promote cell migration and nutrient transport [41]. With constant pore size (600 μm), the scaffolds are designed with different porosity (69%, 74%, 89%). Moreover, the compressive strength of high porosity scaffolds was much lower (0.99 GPa). Therefore, the optimal pore structure of the scaffold was fixed to an average pore size of 600 μm and 74% porosity. Finally, the porous bone scaffold was successfully prepared by soaking PDA. 

### 3.2. Analysis of Fourier Transform Infrared Spectroscopy

**Figure 3:**
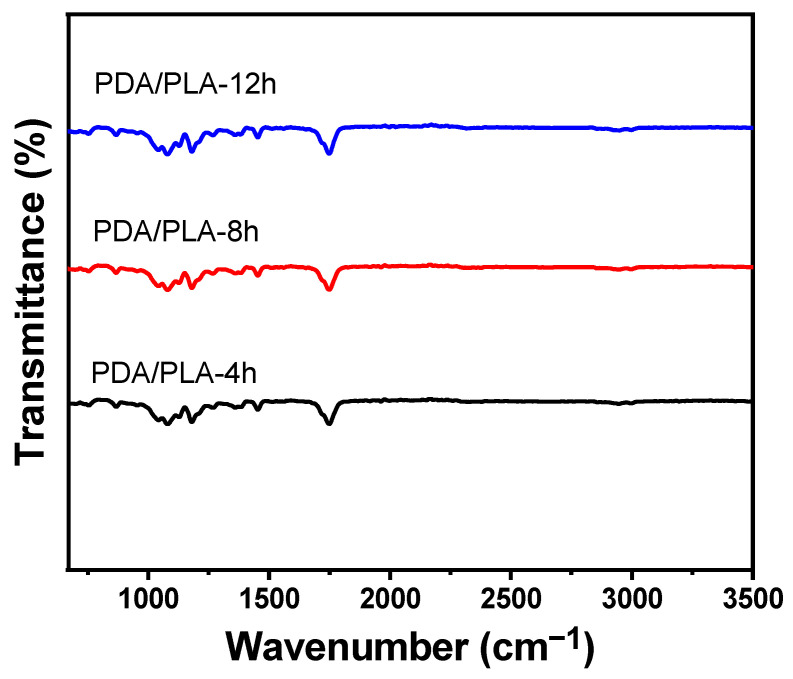
FTIR spectra of PDA/PLA scaffolds with different immersion times in poly(dopamine).

As shown in Figure 1a, the preparation process of PDA/PLA scaffolds was demonstrated. Meanwhile, Figure 1b shows that the surface of PLA/PDA scaffolds became darker as the soaking time increased. It is observed that the white PLA scaffold soaked with too much amine was attached to a black substance, which was formed by the oxidative self-polymerization of dopamine. This phenomenon can be preliminarily identified as poly(dopamine) being coated on the surface of the material. In the FTIR spectrum shown in Figure 3, which demonstrates that a broad -OH stretching band associated with the polymeric hydrogen bonds of PDA, can be observed in the range of 3200–3400 cm^−1^. C-H stretching vibration peaks with -CH_3_ and -CH- can be observed at approximately 2990 cm^−1^. The infrared absorption band of the C=O group of PLA shifts to approximately 1750 cm^−1^. Meanwhile, it is noted that the absorption bands of PDA/PLA are shifted to approximately 1077.20 cm^−1^, which is due to the stretching vibration of single bond H and the stretching vibration of single bond O stretching and C-O-C in the ester-based structure [42]. For the modified PDA/PLA scaffold, new peaks were observed at 1451.43 cm^−1^, which correspond to the characteristic functional groups in PDA [43]. These results indicate that PDA was successfully immobilized on the surface of the PLA scaffold.

### 3.3. Analysis of Crystalline Structure

**Figure 4:**
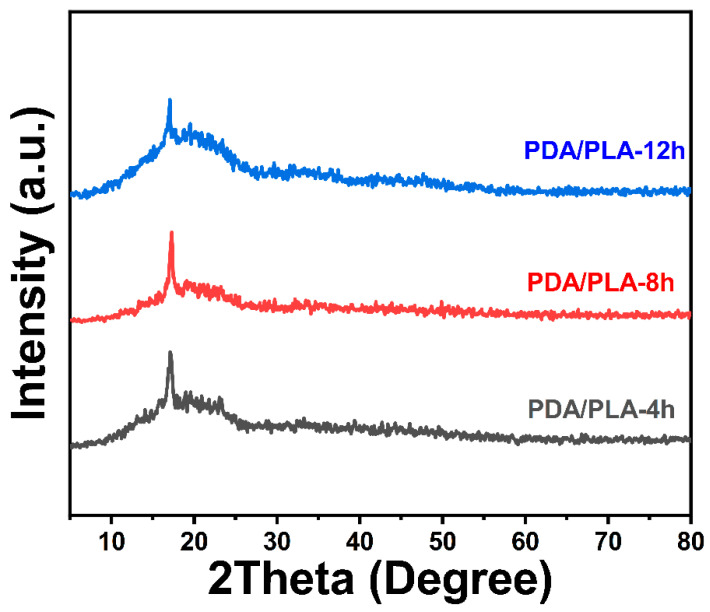
XRD pattern of PDA/PLA scaffolds with different immersion times in poly(dopamine).

Figure 4 of XRD shows that there is no peak corresponding to PDA, which is due to the relatively small amount of dopamine hydrochloride added [44]. However, the diffraction peak shown in the figure is consistent with the crystallization peak of pure PLA at approximately 17°, which can be inferred to be the characteristic peak of PLA [45]. The addition of the PDA coating did not change the crystal structure of PLA, and no new diffraction peaks appeared.

### 3.4. Hydrophilicity

**Figure 5:**
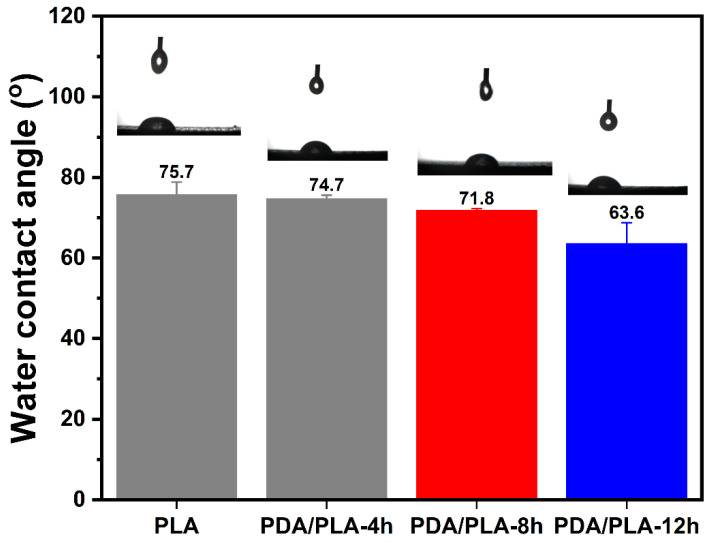
The water contact angle of PDA/PLA scaffolds with different immersion times in poly(dopamine).

Surface wettability has a crucial influence on the initial biological response of scaffolds [46]. The adhesion and proliferation of MSCs could be enhanced on a moderately hydrophilic surface (water contact angle of 40–80°) [47]. However, PLA is a typical hydrophobic polymeric material that lacks cell recognition signals and has limited applications in biomaterials [48]. The structure of poly(dopamine) contains a large number of phenolic hydroxyl and amino functional groups, and the introduction of poly(dopamine) to PLA scaffolds can effectively improve hydrophilicity and bioactivity. In this study, pure PLA material and PDA/PLA material with different coating times were tested for water contact angle, and the results are shown in Figure 5. The results demonstrate that the water contact angle of pure PLA scaffold material is 75.7°, which may be related to the pore structure of the scaffold causing droplet drift. Due to the abundance of phenol hydroxy and amino functional groups of poly(dopamine), the hydrophilicity of the PLA scaffold was enhanced. With the gradual increase in polydopamine coating time, the poly(dopamine) coating on the scaffolds continued to increase, resulting in a gradually decreasing trend of the water contact angle of the scaffolds (75.7° to 63.5°).

### 3.5. Micromorphology

**Figure 6:**
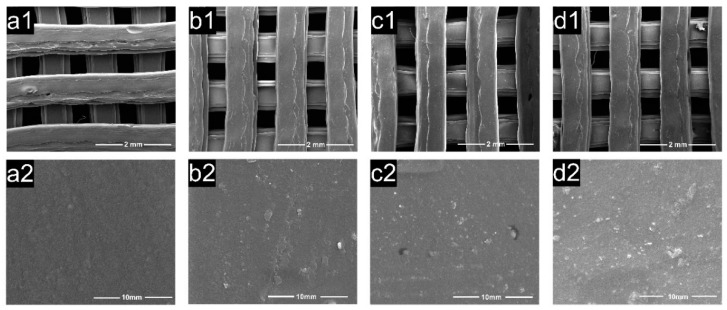
SEM of (**a**) PLA ((**a1**) 60×, (**a2**) 10,000×); (**b**) PDA/PLA-4 h ((**b1**) 60×, (**b2**) 10,000×); (**c**) PDA/PLA-8 h ((**c1**) 60×, (**c2**) 10,000×); (**d**) PDA/PLA-12 h ((**d1**) 60×, (**d2**) 10,000×).

**Figure 7:**
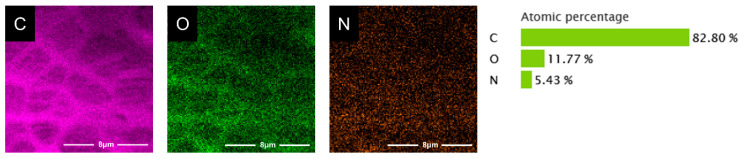
EDS map of C (purple), O (green), and N (orange) atoms on PDA/PLA scaffolds.

The pore structure of polylactic acid scaffolds has an important influence on the adhesion, migration, and proliferation of cells in the human body, and the pore size of polylactic acid scaffolds largely determines whether cells can migrate and grow in scaffolds [41]. Figure 6 exhibits the microscopic images of the samples from SEM, showing the morphology of the PDA/PLA scaffold and the morphology of the hole wall surface with different PDA coating times. The abovementioned figure shows that the scaffold maintained a good porous structure, and the surface morphology of the PDA/PLA scaffold was different from that of the pure PLA scaffold. It can be seen that the coating of PDA made the surface of PLA rough, and the longer dopamine immersion time improved the surface roughness of the scaffold. The rough surface could improve the protein adsorption and cell adhesion ability of the materials [49]. In addition, EDS results in Figure 7 showed that C, O, and N atoms were evenly distributed on the surface of the scaffold, suggesting that PDA was successfully coated on the surface of the scaffold. 

### 3.6. Degradation In Vitro

**Figure 8:**
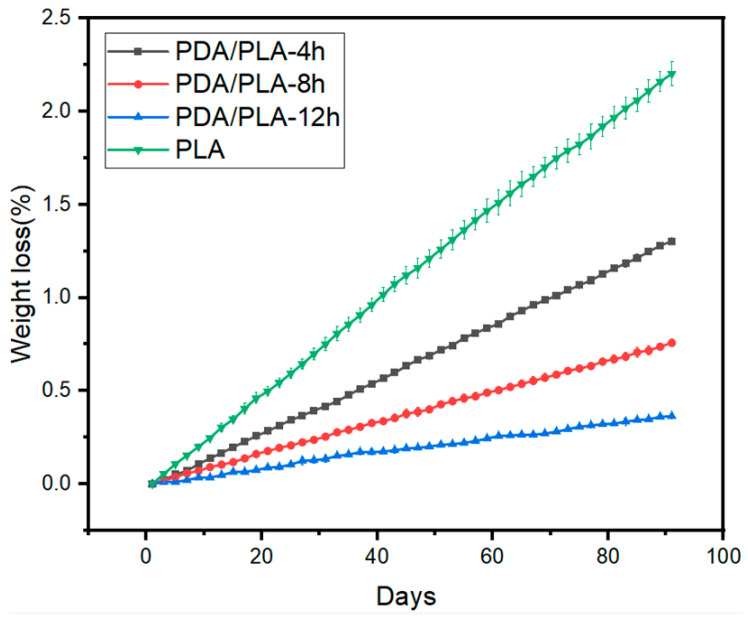
Weight loss of PLA and PDA/PLA samples degradation in PBS buffer.

As a biodegradable polymer, poly (lactic acid) (PLA) is commonly used as a scaffold material for repairing tissue defects. The degradation of pure PLA scaffolds and polydopamine-coated PLA scaffolds with different coating times in pH = 7.4 PBS buffer was recorded. Figure 8 shows that pure PLA scaffolds degraded more in the early stage. Due to the large contact-specific surface area of the porous structure, some small molecules were washed out during the PBS soaking process, and the quality was significantly reduced. However, the hydroxyapatite in the PBS buffer solution will be deposited in the inside of the pores of the scaffold and cannot be completely removed, resulting in an increase in mass, which eventually leads to fluctuations in the weight loss rate of the stent. However, in the later stage, the degradation rate became stable and was maintained at a slower rate. After three months of degradation, the quality of the PLA scaffolds decreased by approximately 2.22%, and the total mass of PDA/PLA-4 h decreased by 1.3%. For PDA/PLA-8 h, it was decreased by 0.75%, while for PDA/PLA-12 h, it was decreased by 0.36%. It can be seen that the poly(dopamine) coating layer delays the degradation of the poly(lactic acid) scaffold to a certain extent due to its refractory degradability. However, it has been previously demonstrated that PDA can be taken up by cells and remain stable in major organ cells for several weeks; thus, the insolubility of PDA does not affect its biocompatibility [50]. Degradation of PLA typically occurs through hydrolytic cleavage of ester bonds. In this case, random chain scission leads to a decrease in molecular weight and a rapid decrease in oligomer and monomer content; finally, complete dissolution of the polymer can be observed. The degradation rate of the composite material prepared in this experiment is maintained at a slow level, and the degradation period may be longer; thus, it can be used to repair bone tissue with a long growth cycle and can effectively avoid the degradation caused by rapid material degradation.

### 3.7. Mineralization In Vitro

**Figure 9:**
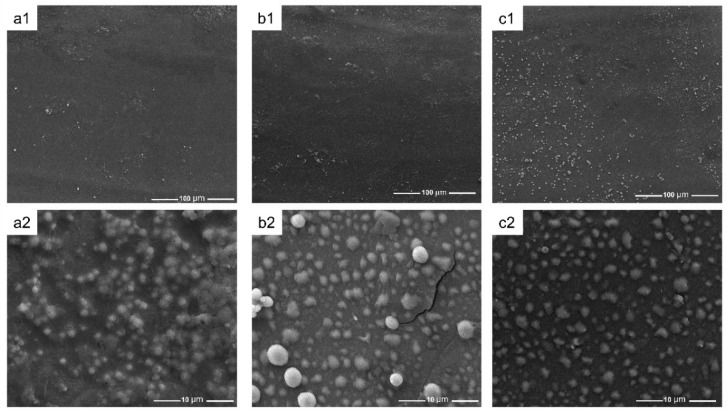
SEM of (**a**) PDA/PLA-4 h ((**a1**) 1000×, (**a2**) 10,000×); (**b**) PDA/PLA-8 h ((**b1**) 1000×, (**b2**) 10,000×); (**c**) PDA/PLA-12 h ((**c1**) 1000×, (**c2**) 10,000×) after 7 days of in vitro mineralization.

**Figure 10:**
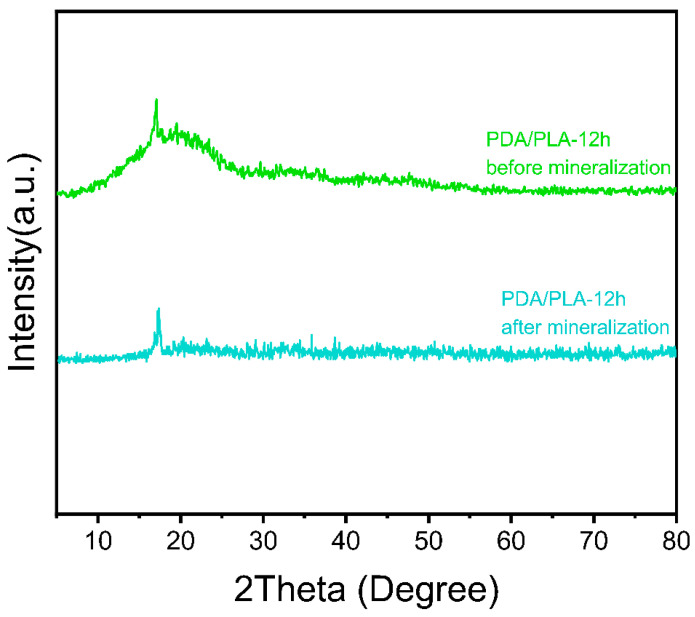
XRD pattern of PDA/PLA-12 h scaffold before and after mineralization.

The ion concentration of simulated body fluid (SBF) is approximately equal to that of human plasma. This PLA/PDA scaffold can form apatite on its surface in the environment of human blood, and this process can be reproduced in SBF. This means that through the formation of mineralized apatite on the SBF surface, the in vivo bioactivity of the material can be predicted [51]. The surface of the porous scaffold shown in Figure 9 demonstrates visible changes after 7 days of SBF soaking, and extensive deposits appeared on the surface of the material. With the increase in the coating time of polydopamine on the surface of the scaffold, the deposition of particles that appeared on the scaffold surface, showed an increasing trend with coating time, which might be hydroxyapatite (HA). In addition, the XRD pattern of PDA/PLA samples after mineralization (Figure 10) showed that the strength of the PLA crystal peak was weakened, which was caused by the degradation of the scaffold and the deposition of estimated HA on the scaffold. It furtherly proved that PDA/PLA scaffold can promote bone deposition sufficiently. It suggests that the formation of apatite can be improved on the surface of the material due to the excellent biological activity and adhesion properties of poly(dopamine).

### 3.8. Near Infrared Response Test

**Figure 11:**
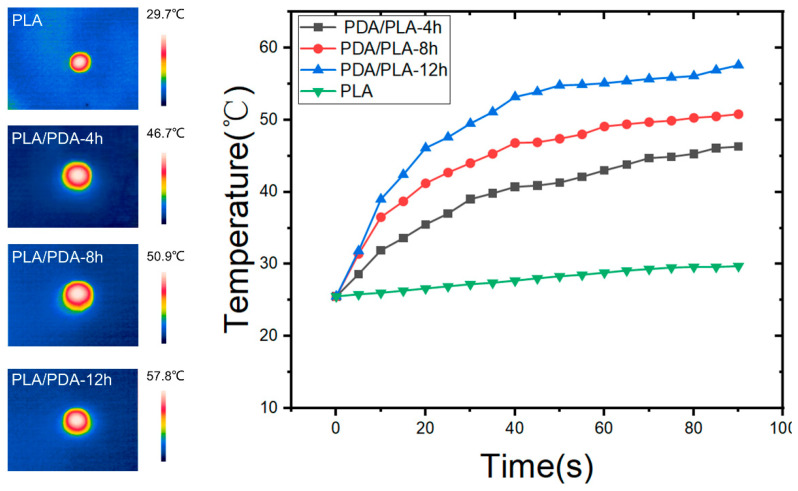
Curves of the critical temperature values of PLA/PDA scaffolds.

In Figure 11, it shows the temperature-changing curves of PLA/PDA scaffolds with different immersion times using NIR laser emitter irradiation (0.2 W/cm^2^) and the critical temperature values are presented. As could be seen, pure PLA samples showed no photothermal effect, other PDA/PLA samples showed a similar trend, i.e., a rapid temperature increase for a relatively short time, followed by a platform of slower heating. When the laser was turned off, the temperature of all samples rapidly cooled in air. With more polydopamine coating, the scaffold heating rate was further accelerated, and the critical temperature of heating was increased from 46.3 °C to 57.6 °C. This efficient photothermal effect is attributed to the uniform coating of PDA on the PLA scaffold. Of note, the temperature of the bone scaffold can be effectively controlled by adjusting the NIR power. It has been demonstrated that the cancer cells can be killed after maintenance at 42 °C for 15–60 min [52], so when conducting photothermal therapy, the heating time and temperature should cause researchers to be wary of causing damage to normal somatic cells. To sum up, the PDA coating makes the PLA/PDA composite scaffold prepared in this work have broad application prospects in the field of photothermal therapy on the basis of excellent biological activity.

## 4. Conclusions

In this study, a light-sensitive biodegradable surface modified PLA scaffold was designed and fabricated using 3D printing and 3D modeling software. The developed PLA scaffolds were designed of an average pore size of 600 μm a porosity of 74%. By soaking the scaffold in dopamine hydrochloride solution and using the self-polymerization reaction of dopamine, the PLA surface was surface functionalized with a polydopamine coating, resulting in a good biological activity of bone engineering. XRD and FTIR results indicated that PDA was successfully coated on the surface of PLA scaffolds. SEM micrographs showed that the surface of the PDA/PLA scaffolds became rough due to the surface coating. After 12 h of PDA immersion, the contact angle of the scaffolds decreased from 75.7° to 63.5°, which shows the improvement of hydrophilicity that can promote cell adhesion. PDA/PLA scaffolds exhibit a tunable photothermal effect under NIR irradiation, and the maximum saturation temperature is 57.8 °C, which means that PDA-coated PLA scaffolds have great potential to be used in photothermal therapy. The 3D-printed PLA/PDA scaffolds have remarkable potential as an alternative material for repairing bone defects.

## Data Availability

No data was used for the research described in the article.

## Abbreviations

PLA	Poly(lactic acid)
PDA	Poly(dopamine)
XRD	X-ray diffraction
FTIR	Fourier transform infrared spectroscopy
SEM	Scanning electron microscopy
EDS	Energy dispersive spectroscopy
WCA	Water contact angle
NIR	Near-infrared
FDM	Fused deposition modeling
FDA	Food and Drug Administration
PTT	Photothermal therapy
PTA	Photothermal agent
SBF	Simulated body fluid

## References

[1] Bose S., Roy M., Bandyopadhyay A. (2012). Recent advances in bone tissue engineering scaffolds. Trends Biotechnol..

[2] Wang L., Zeng X., Yan G., Chen X., Luo K., Zhou S., Zhang P., Li J., Wong T.W. (2021). Biomimetic scaffolds with programmable pore structures for minimum invasive bone repair. Nanoscale.

[3] Ho-Shui-Ling A., Bolander J., Rustom L.E., Johnson A.W., Luyten F.P., Picart C. (2018). Bone regeneration strategies: Engineered scaffolds, bioactive molecules and stem cells current stage and future perspectives. Biomaterials.

[4] Rengier F., Mehndiratta A., von Tengg-Kobligk H., Zechmann C.M., Unterhinninghofen R., Kauczor H.U., Giesel F.L. (2010). 3D printing based on imaging data: Review of medical applications. Int. J. Comput. Assist. Radiol. Surg..

[5] Kulkarni K., Kelderman J., Coleman H., Aguilar M.I., Parkington H., Del Borgo M. (2021). Self-assembly of trifunctional tripeptides to form neural scaffolds. J. Mater. Chem. B.

[6] Zhang S., Xing M., Li B. (2018). Biomimetic Layer-by-Layer Self-Assembly of Nanofilms, Nanocoatings, and 3D Scaffolds for Tissue Engineering. Int. J. Mol. Sci..

[7] Dalton P.D., Klinkhammer K., Salber J., Klee D., Moller M. (2006). Direct in vitro electrospinning with polymer melts. Biomacromolecules.

[8] Chen T., Zou Q., Du C., Wang C., Li Y., Fu B. (2020). Biodegradable 3D printed HA/CMCS/PDA scaffold for repairing lacunar bone defect. Mater. Sci. Eng. C Mater. Biol. Appl..

[9] Serra T., Planell J.A., Navarro M. (2013). High-resolution PLA-based composite scaffolds via 3-D printing technology. Acta Biomater..

[10] Kantaros A., Piromalis D. (2021). Fabricating Lattice Structures via 3D Printing: The Case of Porous Bio-Engineered Scaffolds. Appl. Mech..

[11] Kantaros A. (2022). 3D Printing in Regenerative Medicine: Technologies and Resources Utilized. Int. J. Mol. Sci..

[12] Zein I., Hutmacher D.W., Tan K.C., Teoh S.H. (2002). Fused deposition modeling of novel scaffold architectures for tissue engineering applications. Biomaterials.

[13] Wu W.Z., Geng P., Li G.W., Zhao D., Zhang H.B., Zhao J. (2015). Influence of Layer Thickness and Raster Angle on the Mechanical Properties of 3D-Printed PEEK and a Comparative Mechanical Study between PEEK and ABS. Materials.

[14] Dou Y.C., Huang J.H., Xia X., Wei J.W., Zou Q., Zuo Y., Li J.D., Li Y.B. (2021). A hierarchical scaffold with a highly pore-interconnective 3D printed PLGA/n-HA framework and an extracellular matrix like gelatin network filler for bone regeneration. J. Mater. Chem. B.

[15] Zhang L., Yang G.J., Johnson B.N., Jia X.F. (2019). Three-dimensional (3D) printed scaffold and material selection for bone repair. Acta Biomater..

[16] Chen M., Le D.Q.S., Kjems J., Bunger C., Lysdahl H. (2015). Improvement of Distribution and Osteogenic Differentiation of Human Mesenchymal Stem Cells by Hyaluronic Acid and beta-Tricalcium Phosphate-Coated Polymeric Scaffold In Vitro. BioRes. Open Access.

[17] Naghieh S., Ravari M.R.K., Badrossamay M., Foroozmehr E., Kadkhodaei M. (2016). Numerical investigation of the mechanical properties of the additive manufactured bone scaffolds fabricated by FDM: The effect of layer penetration and post-heating. J. Mech. Behav. Biomed. Mater..

[18] Sa M.W., Nguyen B.N.B., Moriarty R.A., Kamalitdinov T., Fisher J.P., Kim J.Y. (2018). Fabrication and evaluation of 3D printed BCP scaffolds reinforced with ZrO_2_ for bone tissue applications. Biotechnol. Bioeng..

[19] Sood A.K., Ohdar R.K., Mahapatra S.S. (2010). Parametric appraisal of mechanical property of fused deposition modelling processed parts. Mater. Des..

[20] Sood A.K., Ohdar R.K., Mahapatra S.S. (2009). Improving dimensional accuracy of Fused Deposition Modelling processed part using grey Taguchi method. Mater. Des..

[21] Es-Said O.S., Foyos J., Noorani R., Mendelson M., Marloth R., Pregger B.A. (2000). Effect of layer orientation on mechanical properties of rapid prototyped samples. Mater. Manuf. Process..

[22] Feng J. (2017). Preparation and properties of poly(lactic acid) melt spun fiber aligned and disordered scaffolds. Mater. Lett..

[23] Kantaros A. (2022). Bio-Inspired Materials: Exhibited Characteristics and Integration Degree in Bio-Printing Operations. Am. J. Eng. Appl. Sci..

[24] Gandolfi M.G., Zamparini F., Esposti M.D., Chiellini F., Aparicio C., Fava F., Fabbri P., Taddei P., Prati C. (2018). Polylactic acid-based porous scaffolds doped with calcium silicate and dicalcium phosphate dihydrate designed for biomedical application. Mater. Sci. Eng. C Mater. Biol. Appl..

[25] Gremare A., Guduric V., Bareille R., Heroguez V., Latour S., L’Heureux N., Fricain J.C., Catros S., Le Nihouannen D. (2018). Characterization of printed PLA scaffolds for bone tissue engineering. J. Biomed. Mater. Res. Part A.

[26] Kai D., Liow S.S., Loh X.J. (2014). Biodegradable polymers for electrospinning: Towards biomedical applications. Mater. Sci. Eng. C Mater. Biol. Appl..

[27] Zhang Y. (2017). Post-printing surface modification and functionalization of 3D-printed biomedical device. Int. J. Bioprint..

[28] Tsai W.B., Chen W.T., Chien H.W., Kuo W.H., Wang M.J. (2014). Poly(dopamine) coating to biodegradable polymers for bone tissue engineering. J. Biomater. Appl..

[29] Xi D.M., Xiao M., Cao J.F., Zhao L.Y., Xu N., Long S.R., Fan J.L., Shao K., Sun W., Yan X.H. (2020). NIR Light-Driving Barrier-Free Group Rotation in Nanoparticles with an 88.3% Photothermal Conversion Efficiency for Photothermal Therapy. Adv. Mater..

[30] Dong W.J., Li Y.S., Niu D.C., Ma Z., Gu J.L., Chen Y., Zhao W.R., Liu X.H., Liu C.S., Shi J.L. (2011). Facile Synthesis of Monodisperse Superparamagnetic Fe_3_O_4_ Core@hybrid@Au Shell Nanocomposite for Bimodal Imaging and Photothermal Therapy. Adv. Mater..

[31] Huang X.Q., Tang S.H., Mu X.L., Dai Y., Chen G.X., Zhou Z.Y., Ruan F.X., Yang Z.L., Zheng N.F. (2011). Freestanding palladium nanosheets with plasmonic and catalytic properties. Nat. Nanotechnol..

[32] Tian Q.W., Tang M.H., Sun Y.G., Zou R.J., Chen Z.G., Zhu M.F., Yang S.P., Wang J.L., Wang J.H., Hu J.Q. (2011). Hydrophilic Flower-Like CuS Superstructures as an Efficient 980 nm Laser-Driven Photothermal Agent for Ablation of Cancer Cells. Adv. Mater..

[33] Yang K., Zhang S.A., Zhang G.X., Sun X.M., Lee S.T., Liu Z.A. (2010). Graphene in Mice: Ultrahigh In Vivo Tumor Uptake and Efficient Photothermal Therapy. Nano Lett..

[34] Kim M., Boissonnault J.A., Dau P.V., Cohen S.M. (2011). Metal-Organic Framework Regioisomers Based on Bifunctional Ligands. Angew. Chem.-Int. Ed..

[35] Della Vecchia N.F., Avolio R., Alfe M., Errico M.E., Napolitano A., d’Ischia M. (2013). Building-Block Diversity in Polydopamine Underpins a Multifunctional Eumelanin-Type Platform Tunable Through a Quinone Control Point. Adv. Funct. Mater..

[36] Habash R.W.Y., Bansal R., Krewski D., Alhafid H.T. (2006). Thermal therapy, part 1: An introduction to thermal therapy. Crit. Rev. Biomed. Eng..

[37] Liu Y.L., Ai K.L., Liu J.H., Deng M., He Y.Y., Lu L.H. (2013). Dopamine-Melanin Colloidal Nanospheres: An Efficient Near-Infrared Photothermal Therapeutic Agent for In Vivo Cancer Therapy. Adv. Mater..

[38] Gomez S., Vlad M.D., Lopez J., Fernandez E. (2016). Design and properties of 3D scaffolds for bone tissue engineering. Acta Biomater..

[39] Simpson R.L., Wiria F.E., Amis A.A., Chua C.K., Leong K.F., Hansen U.N., Chandraselkaran M., Lee M.W. (2008). Development of a 95/5 poly(L-lactide-co-glycolide)/hydroxylapatite and beta-tricalcium phosphate scaffold as bone replacement material via selective laser sintering. J. Biomed. Mater. Res. Part B Appl. Biomater..

[40] Cavo M., Scaglione S. (2016). Scaffold microstructure effects on functional and mechanical performance: Integration of theoretical and experimental approaches for bone tissue engineering applications. Mater. Sci. Eng. C Mater. Biol. Appl..

[41] Roy T.D., Simon J.L., Ricci J.L., Rekow E.D., Thompson V.P., Parsons J.R. (2003). Performance of degradable composite bone repair products made via three-dimensional fabrication techniques. J. Biomed. Mater. Res. Part A.

[42] Zhou X., Cui H., Nowicki M., Miao S., Lee S.J., Masood F., Harris B.T., Zhang L.G. (2018). Three-Dimensional-Bioprinted Dopamine-Based Matrix for Promoting Neural Regeneration. ACS Appl. Mater. Interfaces.

[43] Xia X., Zhang Y., Chao D., Guan C., Zhang Y., Li L., Ge X., Bacho I.M., Tu J., Fan H.J. (2014). Solution synthesis of metal oxides for electrochemical energy storage applications. Nanoscale.

[44] Guo Z., Zhang D., Qiu H., Ju Y., Zhang T., Zhang L., Meng Y., Wei Y., Chen G. (2016). Improved electrochemical properties of tavorite LiFeSO_4_F by surface coating with hydrophilic poly-dopamine via a self-polymerization process. RSC Adv..

[45] Wang G. (2020). Influence of polydopamine/polylactic acid coating on mechanical properties and cell behavior of 3D-printed calcium silicate scaffolds. Mater. Lett..

[46] Meng C.J., Zhao J.M., Yin Y.X., Luo J., Zhao L.Y., Jiang W.B., Feng J.Y. (2020). Preparation and Characterization of PLA Film/3D Printing Composite Scaffold for Tissue Engineering Application. Fibers Polym..

[47] Luo K., Wang L., Chen X.H., Zeng X.Y., Zhou S.Y., Zhang P.C., Li J.F. (2022). Biocompatible Poly(epsilon-caprolactone)-based Shape-memory Polyurethane Composite Scaffold with Bone-induced Activity. J. Bionic Eng..

[48] Promnil S., Ruksakulpiwat C., Numpaisal P.O., Ruksakulpiwat Y. (2022). Electrospun Poly(lactic acid) and Silk Fibroin Based Nanofibrous Scaffold for Meniscus Tissue Engineering. Polymers.

[49] Papenburg B.J., Rodrigues E.D., Wessling M., Stamatialis D. (2010). Insights into the role of material surface topography and wettability on cell-material interactions. Soft Matter.

[50] Liu X.S., Cao J.M., Li H., Li J.Y., Jin Q., Ren K.F., Ji J. (2013). Mussel-Inspired Polydopamine: A Biocompatible and Ultrastable Coating for Nanoparticles in Vivo. ACS Nano.

[51] Kokubo T., Takadama H. (2006). How useful is SBF in predicting in vivo bone bioactivity?. Biomaterials.

[52] Habash R.W.Y., Bansal R., Krewski D., Alhafid H.T. (2006). Thermal therapy, part 2: Hyperthermia techniques. Crit. Rev. Biomed. Eng..

